# The relationship between food security and quality of life among pregnant women

**DOI:** 10.1186/s12884-018-1947-2

**Published:** 2018-08-06

**Authors:** Farnoosh Moafi, Farideh Kazemi, Fatemeh Samiei Siboni, Zainab Alimoradi

**Affiliations:** 1 Faculty of Nursing and Midwifery, Qazvin University of Medical Science, Qazvin, Iran; 2 Faculty of Nursing and Midwifery, Hamadan University of Medical Science, Hamadan, Iran; 3 Social Determinants of Health Research Center, Qazvin University of Medical Science, Bahonar blv, Qazvin, 34199-15315 Iran

**Keywords:** Food insecurity, Health-related quality of life, Pregnancy

## Abstract

**Background:**

Household food insecurity through influencing the quality and sufficiency of nutrition can have considerable effects on individuals’ health. Previous studies have shown the relationship between household food insecurity and quality of life among adults, infants, and people of minority ethnicity. However, no studies have been conducted on household food insecurity and quality of life among pregnant women. This study aimed to investigate the effect of food insecurity on quality of life among pregnant women in Qazvin city, Iran.

**Methods:**

This cross-sectional study was conducted between May 2017 and November 2017 on 394 pregnant women. A random cluster sampling method was used to select eight urban health and medical centers from four geographical regions of Qazvin city, Iran. In the selected centers, pregnant women were recruited using eligibility inclusion criteria. Data was collected using the SF-36 Health-related Quality of Life, Household Food Insecurity Access Scale and a demographic questionnaire for recording the women’s gestational and demographic information through interviews. Descriptive and inferential statistics including Chi-square test, one-way analysis of variance with Bonferroni post-hoc test and multiple linear regression were used for data analysis. *P* < 0.05 was considered statistically significant.

**Results:**

Food insecurity was reported in 43.9% of the pregnant women. Overall pregnant women’s quality of life had the highest score (Mean ± SD) in the domain of ‘social performance’ (76.4 ± 21) and the lowest one in the domain of ‘role limitation due to physical reasons’ (60.5 ± 43). Pregnant women with food insecurity had the lowest score in role limitation due to physical reasons domain of quality of life (68.6 ± 40.4, 61.3 ± 45.5 & 51.3 ± 47.7 respectively for mild, moderate and sever food insecurity). The results of multiple linear regression showed that one unit reduction of household food security significantly decreased the total quality of life score by 5.2 score (95% CI: -9.7, − 0.7) among the mild food insecure group, 10.8 score (95% CI: -17.1, − 4.6) among the moderate food insecure group and 14.1 score (95% CI: -19.7, − 8.5) among the sever food insecure group.

**Conclusions:**

Screening of the household food security status during the primary prenatal care can identify high-risk pregnant women to improve the quantity and quality of their diet. Moreover multi-level actions including policy-making, supplying resources, and providing appropriate services are needed to ensure that pregnant women have access to high-quality foods.

**Electronic supplementary material:**

The online version of this article (10.1186/s12884-018-1947-2) contains supplementary material, which is available to authorized users.

## Background

Food security is achieved when all people at all times have economic and physical access to sufficient, healthy and nutritious food to meet their dietary needs and food preferences for having an active and healthy life [1]. Limited access to sufficient and safe nutrients or inability to eat appropriate foods through acceptable ways can cause food insecurity [2]. The Food and Agriculture organization (FAO) in the latest report (2018) on the state of food insecurity in almost 150 countries revealed that nearly one in ten individuals in the world (9.3%) suffered from severe food insecurity, which was corresponded to about 689 million individuals. The food security situation visibly has been worsened in sub-Saharan Africa, South Eastern and Western Asia [3]. A recent systematic review on the prevalence of food insecurity in Iran showed that the prevalence of food insecurity was high in Iran. The prevalence of food insecurity was 49% among households, 67% in children, 61% in mothers, 49% in adolescents and 65% in older people [4]. Moreover, according to the 2016’s summary report of food and nutrition security in Iran, the share of food expenditure with reported as 27.1% against the total expenditure, which was not high in Iran. In the food security context, a rate above 65% is considered high food expenditure. According to this report, the food expenditure in Qazvin city was less than 30%, which indicated the food insecurity status [5].

Malnutrition as the most important outcome of food insecurity [6] can result in adverse health consequences in different groups especially women and children [7]. Household food insecurity can have considerable effects on women’s health especially during pregnancy [8]. Nutrition condition during pregnancy not only influences the current health condition of women and infants, but also plays an important role in the health condition of children and adults in the future [9]. Food insecurity has been associated with poor pregnancy outcomes including low birth weight [10], increased risk of congenital defects such as cleft palate, transposition of great vessels, Tetralogy of Fallot and Spina Bifida [11], increased risk of vertical transmission of HIV in HIV-positive women [12] and increased pregnancy-related mortalities [13].

Food insecurity and food shortages are associated with poor general, mental and physical health in women [14]. A study in the USA indicated that food insecurity was associated with women’s reduced mental health [8]. Mental symptoms including depression, stress and anxiety were associated with the household food insecurity in a dose-response relationship, and were increased with worsening the food insecurity status [15]. Also, household food insecurity may reduce quality of life (QoL) [14]. Therefore, the relationship between food insecurity and low QoL among patients with cancer in ethnic minorities [16], in patients with HIV [17] and infants [18] has been reported. Also Stuff et al. (2004) reported that food shortage was associated with low QoL among adults [19].

QoL in Iranian pregnant women has been examined in some studies. Abbaszadeh et al. [20] in a study in Kashan used the SF-36 QoL questionnaire and reported that the lowest QoL score was in ‘functional limitations due to physical health problems’ and ‘vitality’. Some dimensions of health in the SF-36 QoL questionnaire was associated with age, gestational age, gravid, education level and income. In other studies, Azizi et al. [21] reported that the mean scores of QoL in mental and physical subscales of unwanted pregnancies were lower than those with desired pregnancy. Also Jouybari et al. [22] reported that a small percentage of pregnant women had desirable QoL. Despite the importance of household food security and its probable effect on maternal and fetal health during pregnancy, there was no studies on the relationship between household food insecurity and health-related QoL among pregnant women. This research can help clarify the relationship between food insecurity during pregnancy and different domains of health-related QoL. The present study was designed and conducted to investigate the relationship between food insecurity and different Domain of QoL among pregnant women in Qazvin city, Iran.

## Methods

### Study design and settings

This cross-sectional study was conducted between May 2017 and November 2017. All pregnant women referred to the health and medical centers in Qazvin city, Iran were selected. There were 44 health and medical center in this city that provided different types of health care including prenatal care. The coverage of prenatal care was 81.8% in Qazvin city in year 2017. Inclusion criteria included intrauterine pregnancy, gestational age 10–30 weeks, lack of chronic medical problems and gestational complications.

### Sampling

In previous Iranian studies, the prevalence of food insecurity was evaluated using various questionnaires, which varied between 20 and 60% [23]. In the present study, given 50% prevalence of food insecurity, 0.05 precision, and 5% sample loss, the sample size was reported as 403 women.

A random cluster sampling method was used to select urban health and medical centers of Qazvin city. For the purpose of maximum variations in the socioeconomic status, Qazvin city was divided into four geographical regions. From each geographical region, two urban health and medical centers were selected randomly using the table of random numbers. In total, eight urban health and medical centers were chosen. In the selected centers, 40–80 pregnant women were referred monthly. In the selected centers, all pregnant women with eligible inclusion criteria were recruited.

### Definition of variables and measurement

The household food security status, health-related QoL and some gestational and demographic characteristics were investigated in this research. To investigate the household food security status, the Household Food Insecurity Access Scale (HFIAS) was used. This scale reflected the feelings of the head of household about food insecurity of his/her own and the family. In the HFIAS, questions did not refer directly to the nutrition quality, but it covered the household’s perception of changes in food quality, regardless of actual food compositions [24]. The HFIAS was consisted of 9 questions with a 4-item Likert scale as frequently; sometimes; rarely and never. The mentioned responses were scored as 3, 2, 1, 0, respectively. The maximum score for a household was 27. When the household response to all nine questions was “often”, the response score was 3, but the minimum score was 0 when the household responded ‘no’ to all questions. Higher scores in the HFIAS meant the worse status of food insecurity for household. In this scale, food insecurity was divided into four groups including: food secure (0–1 point), mildly food insecure (2–7 points), moderately food insecure (8–14 points) and severely food insecure (15–27 points) [24]. The Farsi version of this questionnaire was carried out by Salarkia et al. (2009) in Varamin city using content-related validity, criterion-related validity and factor analysis. The Chronbach alpha’s coefficient was reported as 0.95 indicating the high internal reliability of the questionnaire [25].

The health-related QoL was measured using the SF-36 QoL questionnaire. The Short Form Health Survey (SF-36) was a well-known health-related QoL instrument that was developed in the USA [26]. The SF-36 was a general QoL instrument that measured eight health related concepts: physical functioning (PF-10 items), role limitations due to physical problems (RP-4 items), bodily pain (BP-2 items), general health perceptions (GH-5 items), vitality (VT-4 items), social functioning (SF-2 items), role limitations due to emotional problems (RE-3 items), and perceived mental health (MH-5 items). Raw scores were transformed to a 0–100 scale with higher scores indicating better QoL [26]. This instrument was translated and validated by Montazeri et al. in 2005 for the first time in Iran. The Farsi version had the Cronbach’s alpha coefficients ranging from 0.77 to 0.90 for subscales. Its validity was examined by the Known groups’ comparison indicating that the SF-36 questionnaire discriminated significantly between men and women, and old and the young respondents as anticipated. In general, the Farsi version of the SF-36 questionnaire was found well and findings suggested that it was a reliable and valid measure of health related QoL among the general population [27].

The demographic characteristics including pregnant women’s age, education level and job, the husband’s education level and job, family living place, living house ownership status and perceived economic status as well as gestational characteristics including current gestational rank, gestational age (week), number of children, pregnancy willingness status and gender of fetus were assessed. The demographic questionnaire was developed according to study objectives through literature search. The validity of this questionnaire was assessed using content validity. Therefore, ten faculty members from midwifery, obstetrics and gynecology departments were asked to assess the questionnaire. Their perspectives led to some revisions. Data collection was performed through interviewing. Sample questionnaires used for data gathering purpose in this study is provided as Additional file 1.

### Statistical analysis

Data was analyzed using the SPSS software version 21 [28]. Descriptive and inferential statistics were used for data analysis. The Kolmogorov-Smirnov test was applied to investigate the normality of quantitative variables to use parametric tests. The relationship between QoL and the household food insecurity status was investigated using the ANOVA test with Bonferroni post-hoc test. The Chi-square test was applied to investigate the relationship between demographic and reproductive characteristics and the food insecurity status. To investigate the effect of household food insecurity on QoL among pregnant women, multiple linear regression via the ENTER method was used. The multiple linear regression model was adjusted for possible confounding factors including demographic and reproductive characteristics which had *p* < 0.2 in the Chi-square test. To apply linear regression, assumptions were tested and data was checked for outliers. In the regression analysis, nominal independent variables with more than two categories were defined as dummy variable. After running the regression model, collinearity was checked, none of the variables had variance inflation factor (VIF) more than 2 and tolerance < 0.1. *P* < 0.05 was considered statistically significant. Raw dataset used and analyzed for this study is provided as Additional file 2.

### Ethical considerations

This study was one part of a research project supported and corroborated ethically by Qazvin University of Medical Sciences, Qazvin, Iran (decree code: IR.QUMS.REC.1394.351). Permissions to enter the health and medical centers were obtained. Next, the researcher introduced himself to the women. After expressing objectives, assuring them about their confidentiality and possibility to withdraw from the study, the written informed consent was signed by the women. Those pregnant women who were willing to participate in this research were interviewed by a well-trained co-researcher to fill out the questionnaires.

## Results

### Demographic characteristics of the women

In this study, 394 pregnant women with the mean age (± SD) of 28.39 ± 5.22 years participated in the present research. The majority of the pregnant women and their husbands had no academic education level (65.2 and 68.3%, respectively). Also, 91.9% were housewives and 5.8% of their husbands were unemployed. Most women lived in urban areas (97.2%), and also the majority of them (47%) had a poor economic status. Most women experienced their first pregnancy. Their mean gestational age was 22.68 ± 5.04 weeks. Furthermore, 82.5% of them had wanted pregnancies. Table 1 provides demographic and gestational characteristics of the women.

**Table 1:** Demographic and gestational characteristics of participants in different levels of food insecurity

variable	Food security status	Food secure (*n* = 221)	Mild food insecure (*n* = 86)	Moderate food insecure (*n* = 37)	Sever food insecurity (*n* = 50)	*p*-value of χ^2^
		No (%)	No (%)	No (%)	No (%)	
Age (year)	25 <	57 (25.8)	17 (19.8)	14 (37.8)	7 (14)	0.02
	25 to 35	139 (62.9)	57 (66.3)	14 (37.8)	33 (66)	
	35<	25 (11.3)	12 (14)	9 (24.3)	10 (20)	
Number of children	No child	130 (58.8)	46 (53.5)	15 (40.5)	21 (42)	0.02
	1	72 (32.6)	25 (29.1)	12 (32.4)	22 (44)	
	2	15 (6.8)	13 (15.1)	7 (18.9)	4 (8)	
	3≤	4 (1.8)	2 (2.3)	3 (8.1)	3 (6)	
Gestational Age (weeks)	< 20	67 (30.3)	23 (26.7)	8 (21.6)	11 (22)	0.17
	≤ 20	154 (69.7)	63 (73.3)	29 (78.4)	39 (78)	
Pregnancy willingness	Wanted	195 (88.2)	67 (77.9)	26 (70.3)	37 (74)	0.006
	Unwanted	26 (11.8)	19 (22.1)	11 (29.11	13 (26)	
Fetus’ gender	Female	92 (41.6)	33 (38.4)	11 (29.7)	21 (39.8)	0.37
	Male	81 (36.7)	30 (34.9)	20 (54.1)	21 (38.6)	
	Don’t known	48 (21.7)	23 (26.7)	6 (16.2)	8 (21.6)	
Women’s educational Status	Under Diploma	129 (58.4)	64 (74.4)	28 (75.7)	40 (80)	< 0.001
	Above Diploma	92 (41.6)	22 (25.6)	9 (24.3)	10 (20)	
Husband’ educational status	Under Diploma	140 (63.3)	65 (75.6)	28 (75.7)	43 (86)	< 0.001
	Above Diploma	81 (36.7)	21 (24.4)	9 (24.3)	7 (14)	
Women’s job	Housewife	202 (91.4)	75 (89.5	35 (94.6)	45 (90)	0.82
	Employed	19 (8.5)	9 (10.5)	2 (5.4)	5 (10)	
Husband’ job	Unemployed	5 (2.3)	8 (9.3)	3 (8.1)	7 (14)	0.004
	Employed	216 (97.7)	78 (97.7)	34 (91.9)	43 (86)	
Residency	Urban	217 (98.2)	84 (97.7)	35 (94.6)	47 (94)	0.30
	Rural	4 (1.8)	2 (2.3)	2 (5.4)	3 (6)	
Residential home status	Landlord	103 (46.6)	40 (46.5)	14 (37.8)	17 (34)	0.33
	Tenant	118 (53.4)	46 (53.5)	23 (62.2)	33 (66)	
Perceived economic Status	Week	97 (43.9)	44 (51.2)	22 (59.5)	31 (62)	0.27
	Moderate	105 (47.5)	35 (40.7)	13 (35.1)	16 (32)	
	Good	19 (8.6)	7 (8.1)	2 (5.4)	3 (6)	
Total		221 (100)	86 (100)	37 (100)	50 (100)	

### Status of household food security and QoL among the women

Regarding household food insecurity among the pregnant women based on the HFIAS scale, the most food insecurity experiences were ‘Eat just a few kinds of foods due to a lack of resources’, ‘Unable to eat preferred foods due to lack of resources’ and ‘Eat foods they really do not want to eat due to impossibility of providing other foods’. Further analysis showed that 21.8% of the pregnant women experienced mild food insecurity, 9.4% moderate food insecurity and 12.7% sever food insecurity. Table 2 showed the prioritization of the food insecurity based on the HFIAS scale as well as the total food insecurity status among the women.

**Table 2:** Prioritization of the food insecurity experiences of the participants and their overall household food security status based on HFIAS

Food insecurity experiences	Frequency of occurrence				Mean (SD)
	Never	Rarely	Sometimes	Frequently	
	No (%)	No (%)	No (%)	No (%)	
Eat just a few kinds of foods due to lack of resources	259 (65.7)	61 (15.5)	58 (14.7)	16 (4.1)	0.57 (0.9)
Unable to eat preferred foods due to lack of resources	279 (70.8)	56 (14.2)	53 (13.5)	6 (1.5)	0.47 (0.8)
Eat foods they really do not want eat due to impossibility of providing other foods	281 (71.3)	79 (20.1)	24 (6.1)	10 (2.5)	0.40 (0.7)
Worry about food	323 (82)	41 (10.4)	25 (6.3)	5 (1.3)	0.27 (0.6)
Eat fewer meals in a day due to lack of food	345 (87.6)	28 (7.1)	17 (4.3)	4 (1)	0.19 (0.6)
Eat a smaller meal due to lack of food	352 (89.3)	30 (7.6)	8 (2)	4 (1)	0.18 (0.5)
No food of any kind in the household	356 (90.4)	25 (6.3)	8 (2)	5 (1.3)	0.14 (0.5)
Go to sleep hungry due to lack of food	363 (92.1)	22 (5.6)	6 (1.5)	3 (0.8)	0.11 (0.4)
Go a whole day and night without eating due to lack of food	368 (93.4)	14 (3.6)	7 (1.8)	5 (1.3)	0.11 (0.5)
Overall food insecurity status	221 (56.1)	86 (21.8)	37 (9.4)	50 (12.7)	1.79 (1.1)

The results of this research showed that the health-related QoL among pregnant women had the highest and lowest scores in the domain of ‘social performance’ with a mean score ± SD of 76.36 ± 21.1 and “role limitation due to physical reasons” with a mean score ± SD of 60.47 ± 43.02. Table 3 showed different domain of QoL.

**Table 3:** Participants’ status in different Domain of QoL

Domain of QOL	Mean (SD)
General health	66.5(16.9)
Physical performance	70.4 (25.5)
Role limitation due to physical reasons	60.5 (43)
Role limitations due to emotional reasons	70 (42)
Physical pain	65 (23.4)
Social performance	76.4 (21.1)
Perceived Mental health	67.5 (18.6)
Fatigue or vitality	64 (23.4)
Total Score of QOL	67.5 (18)

### Association between QoL with the household food security status

The one-way ANOVA test demonstrated that different Domains of QoL had statistically significant differences based on the household food security status. However, only in ‘physical pain’ domain of QoL, no statistically significant difference was observed based on the household food security status. Moreover, severely food insecure pregnant women had significantly the lowest scores of total QoL as well as the lowest scores in almost all domains of QoL. Table 4 showed the mean ± SD of the pregnant women’s QoL based on the household food security status beside the results of the ANOVA test.

**Table 4:** Mean (SD) of the pregnant women’s QoL based on the household food security

		General health	Physical performance	Role limitation due to physical reasons	Role limitations due to emotional reasons	Physical pain	Social performance	Perceived Mental health	fatigue	Total QoL
Household food insecurity status	Food secure (*n* = 221)	70.3 (15.4)	73.4 (24.6)	67.9 (41.3)	76.2 (39.3)	66.2 (22.1)	81.3 (19.1)	70.6 (16.6)	66.4 (16.6)	71.5 (16.2)
	Mild food insecure (*n* = 86)	65.9 (14.7)	69.8 (26)	50.3 (44)	68.6 (40.4)	66.1 (26.9)	75 (19.8)	68 (17.84)	64.7 (19.9)	66.1 (16.7)
	Moderate food insecure (*n* = 37)	57.3 (17.9)	68.1 (21.7)	55.4 (40.5)	61.3 (45.5)	60.6 (19.7)	67.6 (18.5)	58.6 (20.4)	55.7 (22.7)	60.6 (16.8)
	Sever food insecure (*n* = 50)	57.6 (20.1)	60.1 (29)	49 (45.2)	51.3 (47.7)	60.3 (25)	63.5 (25.6)	59.4 (22.4)	55.2 (19)	57.1 (2.5)
One-way ANOVA	F	13.17	3.95	5.31	5.65	1.37	13.65	8.60	7.50	12.28
	*p*-value	< 0.001	0.009	0.001	0.001	0.25	< 0.001	< 0.001	< 0.001	< 0.001

To investigate the effect of the household food insecurity status on QoL, multiple linear regression was applied. The regression model was adjusted for women’s age, women and the spouse education level, number of children, pregnancy willingness and spouse job. The linear regression showed that worsening the household food security could significantly reduce the quality of life among pregnant women. Reducing household food security even for 1 unit significantly decreased the total QoL score by 5.2 scores (95% CI: -9.68, − 0.72) among the mild food insecure group, 10.83 scores (95% CI: -17.08, − 4.58) among the moderate food insecure group and 14.11 scores (95% CI: -19.70, − 8.53) among the sever food insecure group. Decreasing household food security for 1 unit had the greatest impact on “Role limitations due to emotional reasons” domain of QoL by 25.14 scores decrease in the sever food insecure pregnant women. It had the least impact on “Fatigue or vitality” domain of QoL by 0.43 scores decrease in the mild food insecure pregnant women. Table 5 provided the results of adjusted multivariate linear regression between the HFIA score and QoL domains.

**Table 5:** Results of adjusted multivariate linear regression between HFIA score and QOL domains

Household food insecurity status^b^	General health		Physical performance		Role limitation due to physical reasons		Role limitations due to emotional reasons		Social performance		Perceived Mental health		Fatigue or vitality		Total Score of QOL	
	B^a^ (95% CI)	β^a^	B (95% CI)	β	B (95% CI)	β	B (95% CI)	β	B (95% CI)	β	B (95% CI)	β	B (95% CI)	β	B (95% CI)	β
Mild food insecure	− 3.7 (−7.8, 0.5)	− 0.1	− 3.7 (− 11.2, 2.9)	− 0.1	− 18 (− 28.8, − 7.2)	− 0.2	− 7.8 (− 18.5, 2.9)	− 0.1	− 5.8 (− 11, − 0.5)	−0.1	−1.6 (− 6.2, 3.1)	−0.03	− 0.4 (− 5.2, 4.3)	−0.01	−5.2 (− 9.7, − 0.7)	−0.1
Moderate food insecure	−12.1 (− 18, − 6.3)	−0.2	− 5.6 (− 14.7, 3.5)	−0.1	− 13.8 (− 28.9, 1.3)	−0.1	−16.4 (− 31.3, − 1.5)	−0.1	− 13.3 (− 20.6, − 6)	−0.2	− 10.3 (− 16.8, − 3.9)	−0.2	−8.5 (− 15.2, − 1.9)	−0.1	− 10.8 (− 17.1, − 4.6)	−0.2
Sever food insecure	−11.7 (− 16.9, − 6.5)	− 0.2	−13.3 (− 21.4, − 5.1)	− 0.2	− 20 (− 33.5, − 6.5)	− 0.2	− 25.1 (− 38.5, − 11.8)	−0.2	− 16.8 (− 23.3, − 10.3)	−.03	− 9.4 (− 15.2, − 3.7)	− 0.2	−9.5 (− 15.4, − 3.6)	− 0.2	− 14.1 (− 19.7, − 8.5)	−0.3

^a^B and β in all domains and total score of QOL have adjusted for age, women and her spouse educational status, number of children, pregnancy willingness, spouse job

^b^Reference variable: food secure

## Discussion

This study was conducted to investigate the prevalence of food insecurity among pregnant women and find its relationship with different domains of QoL. Food security and the suitable nutrition status are necessary for human’s growth and development, which need access to sufficient, various and high-quality food resources [29].

Regarding, the food insecurity status in the present study, 56.1% of the pregnant women were in a food secure status, but 43.9% of them experienced mild to severe food insecurity. The results of the present study were consistent with those of Sholeye et al. (2014) indicating that 53.6% of urban pregnant women had a food secure status [30]. However, the results of this study were inconsistent with those of Laraia et al. (2006) that 75% of pregnant women were in the food secure status, 15% in marginal insecurity and 10% in the food insecure status [8]. Such differences in results can be due to differences in scales used for investigating the food insecurity status and its effects on identifying the food security status. To evaluate the household food security status, the HFIAS questionnaire was used in the present study, but Sholeye and Laraia employed the USAD 6-item short questionnaire and USAD 18-item questionnaire, respectively. Therefore, due to differences of food insecurity measurement scales, an accurate comparison of the prevalence of gestational food insecurity in different populations is difficult.

The women rather experienced “consumption of a limited amount of food due to lack of resources”, “disability to consume favorable foods due to lack of resources” and “consumption of foods that the family members do not like due to the impossibility of providing other foods”. Accordingly, household food insecurity occurred as the reduced diversity of consumed foods in the family. One of the major outcomes of food insecurity in families is changes in the way of receiving foods as well as in food diversity within the family [23]. Assessment of the food consumption pattern in the households indicates that food insecure households mainly focus on receiving energy or getting the bellies full. For this reason, such families are inclined to consume inexpensive foods with a high energy density, but a low value in terms of micronutrients, diets that are poor in fruits, vegetables, milk and dairies, and food patterns with a low health level [31–33]. Therefore, as the first consequence of insufficient access to food resources, diversity of foods is reduced in such families [2]. This is of special importance among women, because it causes reduction in the consumption of micronutrients among women in reproductive age, considerable reduction in the consumption of fruits and vegetables and considerable increase in irregular food patterns [34]. Consistently, Mathews et al. (2010) reported that women wioth food insecurity used vegetables 8.8 times lesser than those with food security (OR = 8.8 95% CI: 2.6–29.9) [35]. Therefore, paying attention to the reduced diversity of food intake is specifically important for pregnant women, because they need to meet the fetus’s nutritional needs besides their own needs. Lack of a balanced and diverse diet during pregnancy may cause several short- and long-term damage, both in women and infants.

The multiple regression model showed that worsening the household food security status could significantly decrease pregnant women’s QoL. Also, decreasing household food security for 1 unit decreased the total QoL score by 5.2 scores among the mild food insecure group, 10.83 scores in the moderate food insecure group and 14.11 scores among the sever food insecure group. Such findings reflected the extent of household food insecurity in QoL of pregnant women. No research was found on the relationship between the pregnant women’s QoL and the food security status. Therefore, the results of this study were compared with those studies that evaluated this relationship among women or adults. Consistently, in a study conducted to investigate the relationship between household food security and the health status of adults using the SF-12 questionnaire, Stuff et al. (2004) reported that the adults in food insecure households had significantly lower scores in terms of physical and mental health. They found that the association between food security to the general health status was dependent on race. Likelihood of being healthy was 2.08 (95% CI: 1.17, 3.68) for secure and white participants, but it was 0.45 (95% CI: 0.23, 0.87) for insecure white participants [19]. The existence of a relationship between household food insecurity and physical health and mental distress among urban women was reported in the study conducted by Sharkey et al. (2011). Results of separate urban and rural multivariable logistic regression models for fair-to-poor self-rated health indicated that household food insecurity was associated with fair-to-poor general health among rural women (OR = 3.2, 95% CI 2.2, 4.6) but not in urban women (OR = 1.8, 95% CI 0.9, 3.5) [14]. Similarly, the presence of a relationship between food insufficiency and food insecurity with general, physical and mental health has been also reported by Mathews et al. They reported that women with food insecurity had significantly 60% lower chance to have good mental health with (OR = 0.41 95% CI: 0.16–0.73) [35]. Also Laria et al. (2017) reported that psychosocial indicators were higher among food insecure participants in comparison to food secure participants: perceived stress was 2.24 higher (95 %CI: 1.63, 3.08), the likelihood of trait anxiety was 2.14 higher (95% CI: 1.55, 2.96), the likelihood of depressive symptoms was 1.87 higher (95% CI: 1.40, 2.51), and a locus of control attributed to chance was 1.49 higher (1.09, 2.02). However, these groups had lower internal locus of control score (OR = 0.82, 95% CI: 0.60, 1.12), lower mastery score (OR = 0.49, 95% CI: 0.35, 0.68) and lower self-esteem score (OR = 0.52, 95% CI: 0.38, 0.69) [8].

## Limitations

In the present research, sampling was performed in the urban health and medical centers. Also, the majority of the women were living in urban areas. Therefore, the generalization of the results of this study regarding the food security status and QoL to rural pregnant women needs further studies.

## Conclusion

This study was the first one in Iran to investigate the relationship between the health-related QoL during pregnancy and the household food insecurity status. The main findings of the present study showed that the prevalence of food insecurity during pregnancy was considerable. The household food insecurity causes changes in the food consumption pattern and also leads to the reduced food diversity of the family. Furthermore, the household food insecurity has adverse effects on pregnant women’s health related QoL.

It is suggested to perform screening got the household food security status in families during the primary prenatal care to identify those individuals who were high-risk in terms of food insecurity. For those pregnant women who were in the food insecure status, it is proposed to provide food supplement rations or food coupons. Improving the nutritional status especially during pregnancy requires multi-level actions, including policy-making supplying resources and providing appropriate services with the aim of ensuring the pregnant women’s access to various and high-quality foods.

## Additional files

Additional file 1: Sample Questionnaires; Sample materials used for data gathering purpose in this study is provided as additional file 1. (PDF 316 kb)

Additional file 2: Raw dataset: The datasets used and analyzed during the current study is provided as additional file 2. (XLSX 111 kb)

## Abbreviations

ANOVA: Analysis of Variance
HFIAS: Household Food Insecurity Access Scale
HIV: Human Immunodeficiency Virus
QoL: Quality of Life
SPSS: Statistical Package for the Social Sciences
USA: United States of America
