# Supplementary material for: The relationship between food security and quality of life among pregnant women
Source: BMC Pregnancy Childbirth. 2018 Aug 6;18:319. doi: 10.1186/s12884-018-1947-2 (PMC6091190; doi:10.1186/s12884-018-1947-2)
Supplement: Supplementary file 1 — Sample Questionnaires; Sample materials used for data gathering purpose in this study is provided as additional file 1. (PDF 316 kb) [file 12884_2018_1947_MOESM1_ESM.pdf]

Dear Mother

The following questions are related to the research projects entitled "The relationship between food security and quality of life among pregnant women". The project is confirmed by deputy and ethical committee of Qazvin University of Medical Science, Qazvin, Iran. This project is aimed to investigate the impact of household food insecurity on pregnant women's quality of life. Identifying this relation, can be a guide for providing further care to promote women and newborn's health.

Hereby I confirmed that you are free to participate in the project. If you did not like corporate in the project, no change will happen in your routine care. All of data are gathered anonymously, so you can freely answer all of the questions.

Part 1- Demographic and gestational characteristics.

How old are you?	--------- year		
What is your educational status?	Diploma or Under Diploma	Above Diploma	
What is your spouse's educational status?	Diploma or Under Diploma	Above Diploma	
What is your job?	Housewife	Employed	
What is your spouse's job?	Employed	Un employed	
Where do you live?	Urban	Rural	
Do you own home?	Yes	No	
How do you rate your family economic status?	Weak	Moderate	Good
How many children do you have?			
Do you want this pregnancy?	Yes	No	
pregnancy rank			
Gestational age based on last menstrual period or 1st trimester sonogram	-------- week		
Fetus gender	Male	Female	Don't Know

Part 2- Household Food Insecurity Access Scale (HFIAS)

		Never	Rarely (once or twice in the past four weeks)	Sometimes (three to ten times in the past four weeks)	Often (more than ten times in the past four weeks)
1	In the past four weeks, did you worry that your household would not have enough food?				
2	In the past four weeks, were you or any household member not able to eat the kinds of foods you preferred because of a lack of resources?				
3	In the past four weeks, did you or any household member have to eat a limited variety of foods due to a lack of resources?				
4	In the past four weeks, did you or any household member have to eat some foods that you really did not want to eat because of a lack of resources to obtain other types of food?				
5	In the past four weeks, did you or any household member have to eat a smaller meal than you felt you needed because there was not enough food?				
6	In the past four weeks, did you or any household member have to eat fewer meals in a day because there was not enough food?				
7	In the past four weeks, was there ever no food to eat of any kind in your household because of lack of resources to get food?				
8	In the past four weeks, did you or any household member go to sleep at night hungry because there was not enough food?				
9	In the past four weeks, did you or any household member go a whole day and night without eating anything because there was not enough food?				

SF-36 QUESTIONNAIRE

Name:____________________ **Ref. Dr:___________________** **Date: _______**

ID#: _______________ **Age: _______** **Gender: M / F**

Please answer the 36 questions of the **Health Survey** completely, honestly, and without interruptions.

GENERAL HEALTH:
In general, would you say your health is:
- Excellent
- Very Good
- Good
- Fair
- Poor

Compared to one year ago, how would you rate your health in general now?
- Much better now than one year ago
- Somewhat better now than one year ago
- About the same
- Somewhat worse now than one year ago
- Much worse than one year ago

LIMITATIONS OF ACTIVITIES:
The following items are about activities you might do during a typical day. Does your health now limit you in these activities? If so, how much?

Vigorous activities, such as running, lifting heavy objects, participating in strenuous sports.
- Yes, Limited a lot
- Yes, Limited a Little
- No, Not Limited at all

Moderate activities, such as moving a table, pushing a vacuum cleaner, bowling, or playing golf
- Yes, Limited a Lot
- Yes, Limited a Little
- No, Not Limited at all

Lifting or carrying groceries
- Yes, Limited a Lot
- Yes, Limited a Little
- No, Not Limited at all

Climbing several flights of stairs
- Yes, Limited a Lot
- Yes, Limited a Little
- No, Not Limited at all

Climbing one flight of stairs
- Yes, Limited a Lot
- Yes, Limited a Little
- No, Not Limited at all

Bending, kneeling, or stooping
- Yes, Limited a Lot
- Yes, Limited a Little
- No, Not Limited at all

Walking more than a mile
- Yes, Limited a Lot
- Yes, Limited a Little
- No, Not Limited at all

Walking several blocks
- Yes, Limited a Lot
- Yes, Limited a Little
- No, Not Limited at all

Walking one block
- Yes, Limited a Lot
- Yes, Limited a Little
- No, Not Limited at all

Bathing or dressing yourself

◯ Yes, Limited a Lot ◯ Yes, Limited a Little ◯ No, Not Limited at all

PHYSICAL HEALTH PROBLEMS:

During the past 4 weeks, have you had any of the following problems with your work or other regular daily activities as a result of your physical health?

Cut down the amount of time you spent on work or other activities

◯ Yes ◯ No

Accomplished less than you would like

◯ Yes ◯ No

Were limited in the kind of work or other activities

◯ Yes ◯ No

Had difficulty performing the work or other activities (for example, it took extra effort)

◯ Yes ◯ No

EMOTIONAL HEALTH PROBLEMS:

During the past 4 weeks, have you had any of the following problems with your work or other regular daily activities as a result of any emotional problems (such as feeling depressed or anxious)?

Cut down the amount of time you spent on work or other activities

◯ Yes ◯ No

Accomplished less than you would like

◯ Yes ◯ No

Didn't do work or other activities as carefully as usual

◯ Yes ◯ No

SOCIAL ACTIVITIES:

Emotional problems interfered with your normal social activities with family, friends, neighbors, or groups?

◯ Not at all ◯ Slightly ◯ Moderately ◯ Severe ◯ Very Severe

PAIN:

How much bodily pain have you had during the past 4 weeks?

◯ None ◯ Very Mild ◯ Mild ◯ Moderate ◯ Severe ◯ Very Severe

During the past 4 weeks, how much did pain interfere with your normal work (including both work outside the home and housework)?

◯ Not at all ◯ A little bit ◯ Moderately ◯ Quite a bit ◯ Extremely

ENERGY AND EMOTIONS:
These questions are about how you feel and how things have been with you during the last 4 weeks. For each question, please give the answer that comes closest to the way you have been feeling.

Did you feel full of pep?
- All of the time
- Most of the time
- A good Bit of the Time
- Some of the time
- A little bit of the time
- None of the Time

Have you been a very nervous person?
- All of the time
- Most of the time
- A good Bit of the Time
- Some of the time
- A little bit of the time
- None of the Time

Have you felt so down in the dumps that nothing could cheer you up?
- All of the time
- Most of the time
- A good Bit of the Time
- Some of the time
- A little bit of the time
- None of the Time

Have you felt calm and peaceful?
- All of the time
- Most of the time
- A good Bit of the Time
- Some of the time
- A little bit of the time
- None of the Time

Did you have a lot of energy?
- All of the time
- Most of the time
- A good Bit of the Time
- Some of the time
- A little bit of the time
- None of the Time

Have you felt downhearted and blue?
- All of the time
- Most of the time
- A good Bit of the Time
- Some of the time
- A little bit of the time
- None of the Time

Did you feel worn out?
- All of the time
- Most of the time
- A good Bit of the Time
- Some of the time
- A little bit of the time
- None of the Time

Have you been a happy person?
- All of the time
- Most of the time
- A good Bit of the Time
- Some of the time
- A little bit of the time
- None of the Time

Did you feel tired?
- All of the time
- Most of the time
- A good Bit of the Time
- Some of the time
- A little bit of the time
- None of the Time

SOCIAL ACTIVITIES:
During the past 4 weeks, how much of the time has your physical health or emotional problems interfered with your social activities (like visiting with friends, relatives, etc.)?

- All of the time
- Most of the time
- Some of the time
- A little bit of the time
- None of the Time

GENERAL HEALTH:
How true or false is each of the following statements for you?

I seem to get sick a little easier than other people
- ◯ Definitely true ◯ Mostly true ◯ Don't know ◯ Mostly false ◯ Definitely false

I am as healthy as anybody I know
- ◯ Definitely true ◯ Mostly true ◯ Don't know ◯ Mostly false ◯ Definitely false

I expect my health to get worse
- ◯ Definitely true ◯ Mostly true ◯ Don't know ◯ Mostly false ◯ Definitely false

My health is excellent
- ◯ Definitely true ◯ Mostly true ◯ Don't know ◯ Mostly false ◯ Definitely false
